# An early burst of IFN-γ induced by the pre-erythrocytic stage favours *Plasmodium yoelii *parasitaemia in B6 mice

**DOI:** 10.1186/1475-2875-8-128

**Published:** 2009-06-09

**Authors:** Valérie Soulard, Jacques Roland, Olivier Gorgette, Eliane Barbier, Pierre-André Cazenave, Sylviane Pied

**Affiliations:** 1 Unité d'Immunophysiopathologie Infectieuse, Centre National de la Recherche Scientifique (CNRS) URA 1961, Université Paris VI, Institut Pasteur, 25-28 rue du Dr Roux, 75015 Paris, France; 2 UR010, Santé de la mère et de l'enfant en milieu tropical, Institut de Recherche pour le Développement (IRD), Université Paris Descartes, Faculté de Pharmacie, 4 avenue de l'Observatoire, 75270 PARIS Cedex 06, France; 3 U547, Institut National de la Santé et de la Recherche Médicale (INSERM), Institut Pasteur de Lille, 1 rue du Pr. Calmette, BP 245, 59019 LILLE Cedex, France; 4 Unité d'Immunologie Moléculaire des Parasites, Institut Pasteur, 25-28 rue du Dr Roux, 75015 Paris, France; 5 UPMC-CNRS 7087, Université Pierre et Marie Curie, GH La Pitié Salpétrière, 83 Boulevard de l'Hôpital, 75651 Paris Cedex 13, France

## Abstract

**Background:**

In murine models of malaria, an early proinflammatory response has been associated with the resolution of blood-stage infection. To dissect the protective immune mechanims that allow the control of parasitaemia, the early immune response of C57BL/6 mice induced during a non-lethal plasmodial infection was analysed.

**Methods:**

Mice were infected with *Plasmodium yoelii *265BY sporozoites, the natural invasive form of the parasite, in order to complete its full life cycle. The concentrations of three proinflammatory cytokines in the sera of mice were determined by ELISA at different time points of infection. The contribution of the liver and the spleen to this cytokinic response was evaluated and the cytokine-producing lymphocytes were identified by flow cytometry. The physiological relevance of these results was tested by monitoring parasitaemia in genetically deficient C57BL/6 mice or wild-type mice treated with anti-cytokine neutralizing antibody. Finally, the cytokinic response in sera of mice infected with parasitized-RBCs was analysed.

**Results:**

The early immune response of C57BL/6 mice to sporozoite-induced malaria is characterized by a peak of IFN-γ in the serum at day 5 of infection and splenic CD4 T lymphocytes are the major producer of this cytokine at this time point. Somewhat unexpected, the parasitaemia is significantly lower in *P. yoelii*-infected mice in the absence of IFN-γ. More precisely, at early time points of infection, IFN-γ favours parasitaemia, whereas helping to clear efficiently the blood-stage parasites at later time points. Interestingly, the early IFN-γ burst is induced by the pre-erythrocytic stage.

**Conclusion:**

These results challenge the current view regarding the role of IFN-γ on the control of parasite growth since they show that IFN-γ is not an essential mediator of protection in *P. yoelii*-infected C57BL/6 mice. Moreover, the mice parasitaemia is more efficiently controlled in the absence of an early IFN-γ production, suggesting that this cytokine promotes parasite's growth. Finally, this early burst of IFN-γ is induced by the pre-erythrocytic stage, showing the impact of this stage on the immune response taking place during the subsequent erythrocytic stage.

## Background

Malaria is initiated when sporozoites are injected into the mammalian host during the blood meal of an infected mosquito. The sporozoites reach the liver where they mature and divide within hepatocytes, thus completing the first phase of the parasite life cycle, the pre-erythrocytic stage. When the hepatic forms are mature, thousands of merozoites are released, reach the blood stream and invade red blood cells, initiating the erythrocytic stage. Parasitized RBCs (pRBCs) subsequently release new merozoites, which perpetuate the erythrocytic cycle by invading new RBCs.

In mice, as well as in humans, early immune events play a determinant role in the outcome of malaria, which depends, in part, on a subtle balance between pro- and anti-inflammatory responses. An early proinflammatory immune response has not only been associated with protection, through its contribution to parasite elimination, but also with the severe complications of the disease [1-3].

In murine models of malaria, the early production of IL-12, IL-18, TNF and IFN-γ has been associated with the resolution of blood-stage infection [1,4-6]. Particularly, an early IFN-γ production was shown to be essential to control parasitaemia and to be associated with a better survival prognostic [7-9]. IFN-γ indeed promotes protective Th1 T cell responses *in vivo*, and favours the Th1-associated IgG2a response involved in the clearance of blood-stage parasites [9].

Recently, NK, NKT, and γδT cells were shown to produce IFN-γ during the blood-stage of murine malaria and also in response to *P. falciparum*-infected RBCs *in vitro *[3,10-12].

To better define the early immune mechanisms that promote the control of *Plasmodium *growth and its elimination during primary infection, the non-lethal malaria model of B6 mice infected with *P. yoelii *265BY sporozoites, the natural invasive form of the parasite, was used. Such an experimental condition allows the development *in vivo *of the full *Plasmodium *life cycle that is closer to the natural infection.

In this model, the production of Th1 cytokines *in vivo *was first analysed at early post-infection (p.i.) time points. This showed that *P. yoelii *265BY infection in B6 mice is characterized by a peak of IFN-γ in the serum at day 5 p.i., while concentrations of circulating TNF and IL-12 stayed at the levels found in non-infected animals. Flow cytometric analyses revealed that splenic NKT, NK, γδ and CD4 T cells simultaneously produced this cytokine at day 5 p.i., the latter ones being the major producer. Then, B6.IFN-γ^-/- ^mice were used to evaluate the physiological relevance of this early IFN-γ production on the control of infection *in vivo*. Surprisingly, the parasitaemia of infected B6.IFN-γ^-/- ^was significantly lower than the one of B6 mice. These results were confirmed by *in vivo *neutralization of the early production of IFN-γ in infected B6 mice using anti-IFN-γ Abs. Finally, the stage-specificity of this early IFN-γ burst was addressed and, following infection of B6 mice with *P. yoelii*-infected RBCs, no peak of IFN-γ was detectable in their serum during the first week of infection.

In conclusion, these results show that, during a primary infection initiated with *P. yoelii *sporozoites, IFN-γ plays two opposite roles in the control of parasitaemia and that, unexpectedly, B6 mice control their parasitaemia better in the absence of an early IFN-γ production. Together with a recent report from Couper *et al *[13], the results presented here challenge the current view regarding the role of IFN-γ on the control of the parasite growth and suggest that an early peak of circulating IFN-γ promotes *Plasmodium*'s growth during a primary infection. In addition to that, these data show that the immune response induced by the pre-erythrocytic stage impacts on the control of the subsequent erythrocytic stage. These results highlight the importance of studying the immune response to *Plasmodium *in models as close as possible to the physiology of the natural infection.

## Methods

### Mice

C57Bl/6N@Ico (referred to as B6) mice were purchased from Charles River-Iffa Credo (St-Aubin les Elbeufs, France). CD1d.1^-/-^, IFN-γ^-/- ^and RAG2^-/- ^mice on a C57BL/6 genetic background were provided by A. Bendelac [14], J.F. Bureau (mice bred at the Institut Pasteur, originally from the Jackson Laboratory, Bar Harbor, Maine, USA), and J.P. Di Santo [15] respectively (referred to as B6.CD1d^-/-^, B6. IFN-γ^-/- ^and B6.RAG2^-/-^). All animals were housed and bred in the animal facilities of the Institut Pasteur (Paris, France) under standard conditions. Only 8- to 12-week-old females were used and experiments were conducted in accordance with institutional guidelines for animal care and use.

### Parasites, *in vivo *infection, and parasitaemia

Sporozoites of the uncloned line of the 265BY strain of *Plasmodium yoelii yoelii *were obtained by dissecting the salivary glands of infected *Anopheles stephensi *mosquitoes as previously described [16]. The mosquitoes were bred, maintained, and infected at the CEPIA (Centre de Production et Infection des Anophèles, Institut Pasteur, Paris, France). Mice were infected intravenously with 4,000 sporozoites diluted in sterile PBS or intraperitoneally with 10^6 ^parasitized-RBCs (pRBCs). Parasitaemia was measured by flow cytometry following a protocol adapted from Jouin *et al *[17,18], and Lee *et al *[19]. Briefly, 3 μl of blood taken from the tail vein were fixed in 500 μl of PBS-glutaraldehyde 0.25% (grade I, ref. G5882, Sigma Aldrich, Lyon, France) and stored at 4°C until use. Once all the samples were collected, 50 μl of each were incubated in 400 μl of PBS containing 0.5 mM Hoechst (Bisbenzimide H33258, ref. B 2883, Sigma Aldrich) and 0.1 mg/ml Thiazole Orange (ref. 39 006.2, Sigma Aldrich) for 1 hour, at RT, in the dark. Analysis of the staining was performed on a LSR cytometer using the CellQuest Pro software (BD Biosciences, San Diego, California, USA). Results are expressed as the percentage of pRBCs.

### Isolation of hepatic and splenic mononuclear cells

Hepatic mononuclear cells were prepared as previously described [20]. Briefly, livers from control and infected mice were perfused *in situ *with sterile DMEM, removed and homogenized using a Potter-Elvehjem homogenizer. Cells were washed, resuspended in a 35% Percoll solution (Pharmacia Biotech, Uppsala, Sweden) and centrifuged at 1400 g for 25 min at room temperature (RT). The pellet (containing mononuclear cells) was washed with DMEM. Spleens from control and infected mice were gently smashed between two glass slides in sterile D-MEM (Gibco Invitrogen, Cergy Pontoise, France). The cell suspensions were then washed with D-MEM before erythrocyte lysis with an ACK lysing buffer (0.15 M NH_4_Cl, 10 mM KHCO_3_, 0.1 mM Na_2_EDTA). Finally, cells were resuspended in sterile RPMI 1640 medium + GlutaMAX I (Gibco Invitrogen) containing 3% FCS before counting of living cells in eosin.

### Intracellular cytokine staining for flow cytometric analysis

Splenic cells from control and infected mice were isolated and incubated at a concentration of 1 × 10^6 ^cells per ml, for 1 hour at 37°C in a 5% CO_2 _atmosphere, in RPMI 1640 medium + GlutaMAX I containing 10% FCS, penicillin-streptomycin (100 IU/ml, Gibco Invitrogen), and brefeldin A (10 μg/ml, Sigma Aldrich). Cells were then washed once in PBS-3% FCS and surface antigens were stained at 4°C, in the dark, for 20 min, using the following monoclonal antibodies purchased from BD Biosciences and conjugated to biotine, FITC, PE, APC or PE-cyanine7: anti-NK1.1 (PK136), anti-TCRγδ (GL3), anti-CD3ε (145-2C11), anti-CD4 (L3T4), anti-CD8α (Ly-2). Biotinylated mAbs were revealed with streptavidine-PE-cyanine7 (BD Biosciences). Then, cells were fixed for one hour at RT, in the dark, with 2% PFA, and subsequently treated with Perm/Wash solution (BD Biosciences) before incubation with APC-conjugated anti-IFN-γ (XMG1.2, BD Biosciences) or isotype-matched control mAb (rat IgG1, BD Biosciences) in Perm/Wash solution at RT, in the dark, for 30 min. Finally, cells were washed in Perm/Wash solution and then in PBS-3% FCS. Stained cells were analysed on a six-color LSR flow cytometer with the CellQuest Pro software (BD Biosciences, San Diego, CA).

### Quantification of cytokines in the sera and in culture supernatants by ELISA

Mice were bled at the indicated days and sera were aliquoted and stored at -20°C until use. The mouse IFN-γ ELISA Set (Cat. No. 555138), mouse TNF (Mono/Mono) ELISA Set (Cat. No. 555268), and mouse IL-12p40 ELISA Set (Cat. No. 555165) from BD Biosciences were used to quantify each cytokine following the manufacturer's instructions. Duplicate serial dilutions were performed for each serum, and DO means were used to determine the concentrations of cytokines in the samples according to the standard.

For quantification of IFN-γ in culture supernatants, total splenocytes and iHLs were isolated, resuspended at a concentration of 1 × 10^6 ^cells/ml in complete RPMI 1640 GlutaMAX I (10% FCS, 100 IU/ml penicillin-streptomycin) and distributed at 200 μl/well in 96-well tissue culture plates. Duplicate cultures were done for each sample. After 72 hours of culture, plates were centrifuged, 150 μl of supernatants were collected and stored frozen at -20°C until use. DO means of duplicate wells were used to determine the concentration of IFN-γ in the samples according to the standard.

### *In vivo *treatment of C57Bl/6 mice with anti-IFN-γ mAb

Mice were infected with 4,000 sporozoites and, on the indicated day, were injected intraperitoneally with 2 mg of anti-IFN-γ mAb (XMG1.2, purified from ascites fluid by DEAE cellulose) or with 2 mg of irrelevant control rat IgG (ref. I4131, Sigma).

### Statistical analyses

Statistical analyses were performed using the non-parametric Mann-Whitney test and Statview 5.0 software (SAS Institute Inc., Cary, NC). A p-value < 0.05 was considered significant.

## Results

### *Plasmodium yoelii *sporozoite infection induces an early peak of IFN-γ in the serum of B6 mice

The *in vivo *production of three Th1-type cytokines, namely IFN-γ, TNF and IL-12, during *P. yoelii *primary infection in B6 mice was first adressed. For that purpose, the concentrations of these cytokines in the sera of non-infected and infected mice were determined by ELISA, between day 3 and day 10 post-injection of sporozoites. TNF and IL-12 concentrations did not change during the course of infection and remained at the level found in non-infected control mice.

**Figure 1:**
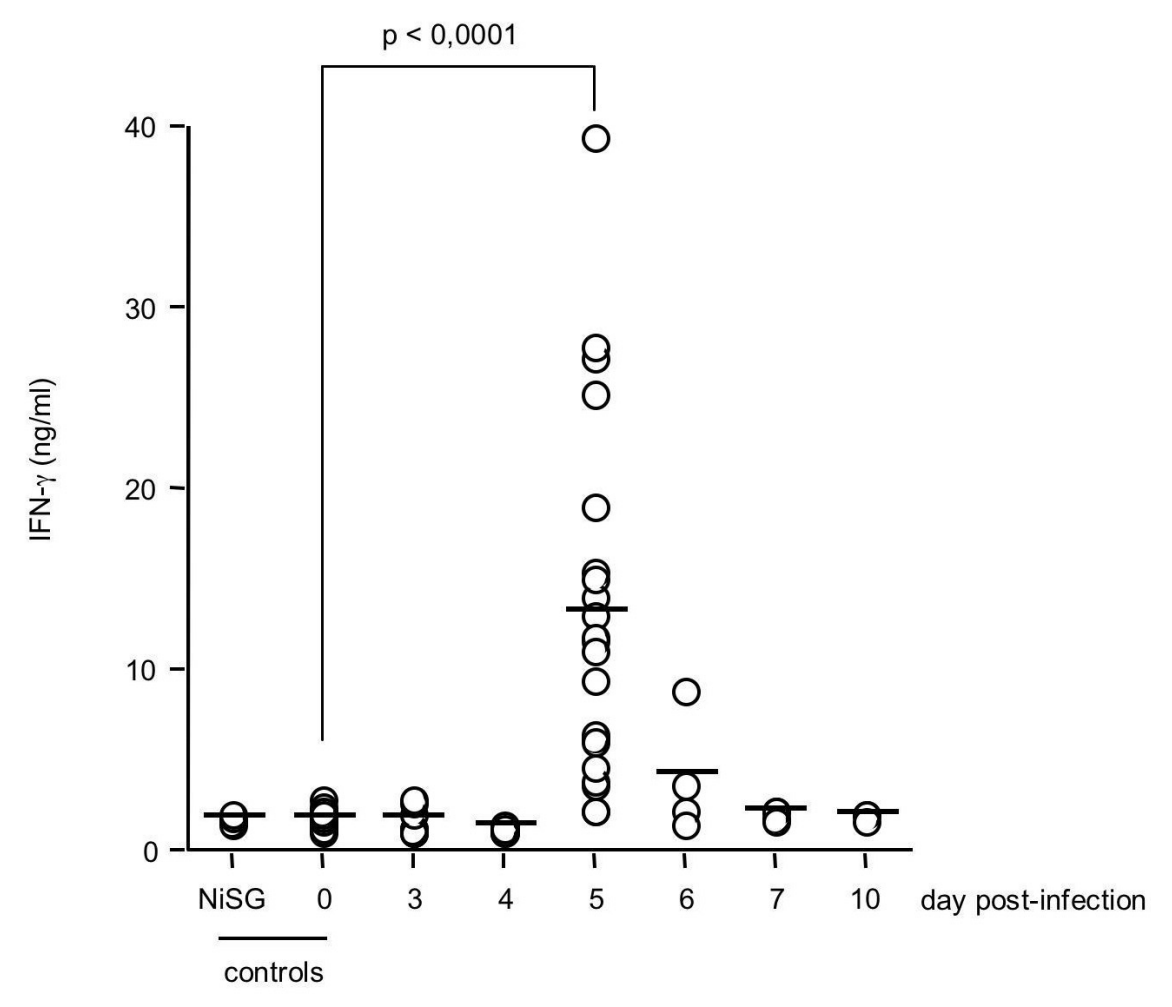
**Primary infection of B6 mice with *P. yoelii *sporozoites is characterized by a peak of IFN-γ in the sera at day 5 post-infection**. B6 mice were infected with 4,000 sporozoites of *P. yoelii *265BY and the serum level of IFN-γ at days 0, 3, 4, 5, 6, 7, and 10 p.i. was determined by ELISA. Results from a pool of 6 independent experiments (3 to 21 mice per time point) are shown. Individual values (circles) and mean values (bars) are shown. NiSG: day-5 serum from B6 mice which received an injection of non-infected salivary gland extract. The statistical difference between day 0 (n = 17) and day 5 p.i. (n = 21) was calculated with the Mann-Whitney test.

Conversely, the concentration of IFN-γ peaked at day 5 p.i. (Figure 1; median value at day 5 p.i. = 11.51 ng/ml, n = 21; median value in non-infected control mice = 1.66 ng/ml, n = 22; p < 0.0001). A large range of IFN-γ concentrations at day 5 p.i. was observed among mice, which could be explained by the transient nature of IFN-γ production. No increase in the IFN-γ concentration was detected in the sera of mice 5 days after injection of salivary gland extracts from non-infected mosquitoes (NiSG, Figure 1). This result shows that the peak of IFN-γ observed at day 5 p.i. is not the result of a non-specific immune response induced by salivary gland extracts, but is specifically induced by *P. yoelii *infection. This peak of IFN-γ was absent from the sera of B6.IFN-γ^-/- ^mice. Thus, *P. yoelii *primary infection in B6 mice, induced by the injection of sporozoites, is characterized by a peak of IFN-γ in the blood at day 5 of infection.

### The early peak of IFN-γ in the serum is dependent on T and/or B lymphocytes, but independent of CD1d-restricted NKT cells

**Figure 2:**
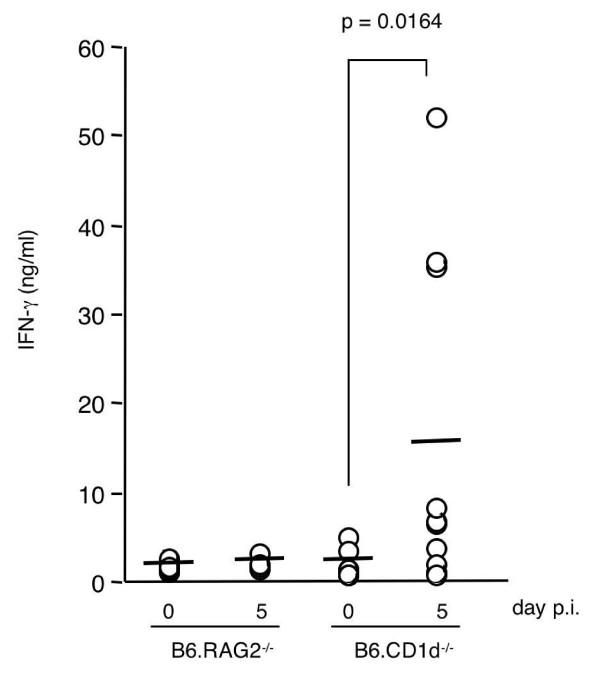
**The early peak of IFN-γ in the serum of *P. yoelii*-infected mice is dependent on T and/or B lymphocytes, but independent of CD1d-dependent NKT cells**. B6.RAG2^-/- ^mice and B6.CD1d^-/- ^mice were infected with 4,000 sporozoites of *P. yoelii *265BY and the serum level of IFN-γ at days 0 and 5 p.i. was determined by ELISA. Results from a pool of 2 to 3 independent experiments per mouse strain (4 to 17 mice per time point) are shown. Individual values (circles) and mean values (bars) are shown. The statistical difference between day 0 (n = 8 B6.CD1d^-/-^) and day 5 p.i. (n = 10 B6.CD1d^-/-^) was calculated with the Mann-Whitney test.

To identify the lymphoid cell populations involved in this early IFN-γ production, sera of B6.RAG2^-/- ^mice (which lack T, B, and NKT cells, but possess NK cells) were tested by ELISA. No peak of IFN-γ was detected in the sera of these mice between day 0 and day 10 p.i. (Figure 2), demonstrating that this early production of IFN-γ is dependent on T, and/or B, and/or NKT lymphocytes. Moreover, these results suggest that NK cells alone cannot initiate the production of this cytokine at a level detectable in the serum.

Since this burst of IFN-γ occurs early after parasite injection, it was postulated that other innate lymphocytes could be the source of this cytokine. In this context, involvement of CD1d-restricted NKT cells, a population of lymphocytes involved in early immune responses to several pathogens, including Plasmodium [21-23], was tested. B6.CD1d^-/- ^mice, which lack CD1d-restricted NKT cells, were infected and IFN-γ in serum was quantified by ELISA at different time points p.i. As shown in Figure 2, the IFN-γ concentration in the sera of B6.CD1d^-/- ^mice increased at day 5 p.i. and reached a similar amount to that detected in day 5-infected B6 mice (B6.CD1d^-/- ^median value at day 5 p.i. = 6.760 ng/ml, n = 10; B6 median value at day 5 p.i. = 11.51 ng/ml, n = 21; p = 0.3932). At days 3, 4, 6, and 7 p.i., IFN-γ concentrations determined in the sera of B6.CD1d^-/- ^mice were similar to those found in non-infected mice. Thus, these results show that CD1d-restricted NKT cells are not necessary for the initiation of the IFN-γ burst detected in the serum at day 5 p.i.

### Splenocytes are the major source of IFN-γ at early time points of *P. yoelii *infection

**Figure 3:**
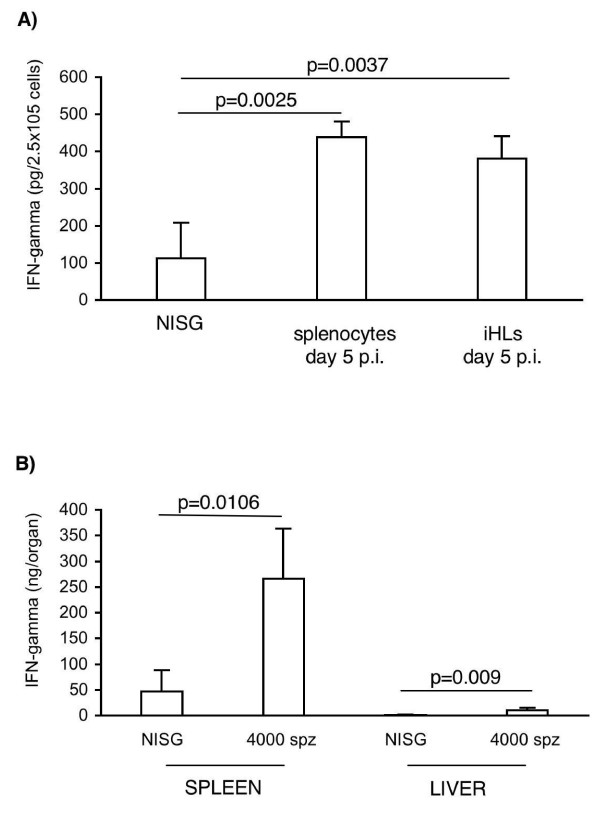
**Splenocytes are the major source of IFN-γ *ex vivo***. B6 mice were either infected with 4,000 sporozoites of *P. yoelii *(n = 10 mice) or received an injection of salivary gland extracts from non-infected mosquitoes (n = 10 NiSG control mice). Five days later, splenocytes and iHLs were isolated and cultured for 3 days without any stimulation. Then, supernatants were collected and IFN-γ concentrations were determined by ELISA. A. Results are expressed as mean values ± SD of IFN-γ in pg per 2.5 × 10^5 ^cells. B. Results are expressed as mean values ± SD of IFN-γ in ng per organ (meaning per total number of splenocytes or iHLs at the indicated days p.i.). Data from a representative experiment out of two are shown.

Since it has previously been observed that the innate immune response to *P. yoelii *infection was compartimentalised and differed between spleen and liver [11,12], the contribution of splenocytes and iHLs to this production of IFN-γ in B6 mice was analysed at days 0 and 5 pi. Splenic and hepatic cells were isolated and IFN-γ was quantified in supernatants after 72 h of culture without any stimulation. As shown in Figure 3A, the concentration of IFN-γ was significantly increased in supernatants of both splenocytes and iHLs isolated from day 5-infected B6 mice. In addition, splenocytes and iHLs produced comparable amounts of IFN-γ for a similar number of cells per culture well (Figure 3A). However, the number of total splenocytes at day 5 of infection was about 60 times higher than the one of total iHLs. So, considering the IFN-γ production that can be attributed to each organ, the spleen appeared to be the main source of IFN-γ (Figure 3B). Consequently, further analyses were focussed on this organ.

### Splenic conventional T CD4 lymphocytes are the major producers of IFN-γ at day 5 p.i

**Figure 4:**
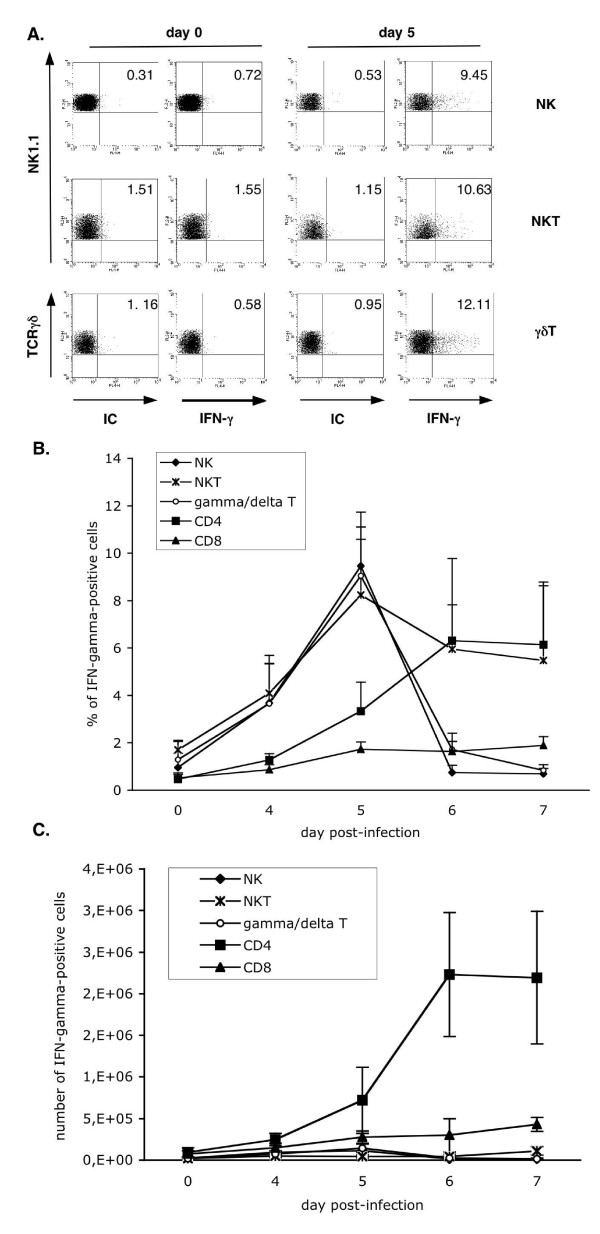
**Splenic NK, NKT, γδT and CD4 T lymphocytes produce IFN-γ simultaneously at day 5 p.i**. Splenocytes were isolated from non-infected and infected B6 mice at days 4, 5, 6, and 7 after injection of 4,000 *P. yoelii *265BY sporozoites. Intracellular expression of IFN-γ among NK cells (gated on NK1.1^+^CD3^-^), NKT cells (gated on NK1.1^+^TCRβ^+^), γδT cells (gated on CD3^+^TCRγδ^+^), CD4 T cells (gated on CD4^+^) and CD8 T cells (gated on CD8^+^) cells was analysed by flow cytometry. A. FACS dot plots showing IFN-γ expression among splenic NK, NKT and γδT cells at days 0 and 5 p.i. IC: isotype-matched control antibody. Percentages of positive cells are indicated. B and C. Kinetics of intracellular IFN-γ expression among the different subsets of lymphocytes. Analysis was performed as shown in A. Results are expressed as mean values ± SD of the percentages (B) or the numbers (C) of IFN-γ-positive cells among each subset. Data are representative of two independent experiments with at least three mice per time point.

In order to precise the cellular source of IFN-γ among splenocytes, the intracellular production of this cytokine by splenic NK cells, NKT cells, γδ CD4 and CD8 T cells during infection was analysed by flow cytometry. Examples of FACS analysis for minor lymphocyte populations are shown in Figure 4A. As shown in Figure 4B, the proportion of IFN-γ-positive cells among NKT, NK, and γδT cells peaked at day 5 p.i., matching with the peak of this cytokine detected in the sera. It can also be noted that the frequency of IFN-γ-positive cells decreases very rapidly after day 5 of infection, except for NKT cells. In parallel, the percentage of IFN-γ-positive conventional CD4T cells started to increase from day 5 p.i. and was maintained stable, at least, until day 7 p.i. (Figure 4B).

However, the number of splenic CD4 T cells secreting IFN-γ at day 5 p.i. was 2.4 and 2.5 times superior to the numbers of IFN-γ-positive innate lymphocytes and CD8 T cells, respectively (Figure 4C). Thus, the production of IFN-γ by splenic innate lymphocytes is simultaneous and peaks at day 5 p.i., but conventional CD4 T cells represent the major source of this cytokine at this time point.

### Absence of the early production of IFN-γ associates with a better control of parasitaemia

**Figure 5:**
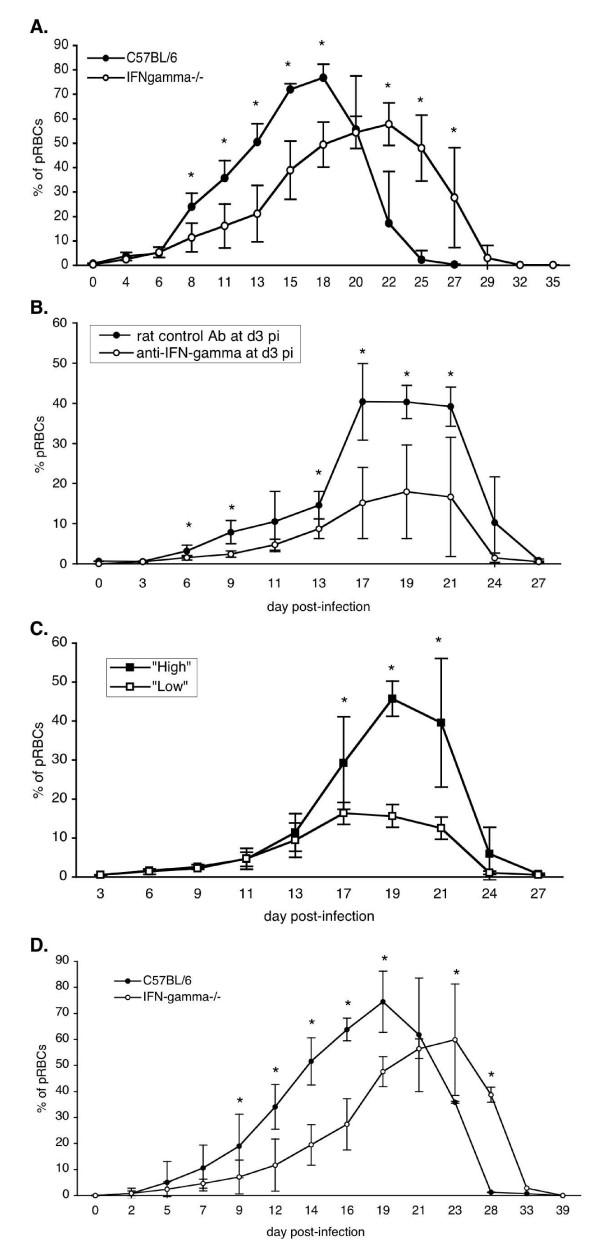
**Parasitaemia of B6 mice is lower in the absence of IFN-γ during primary *P. yoelii *infection**. A. Parasitaemia of B6 control mice and B6.IFN-γ^-/- ^mice following injection of 4,000 sporozoites of *P. yoelii *265BY. B. Parasitaemia of B6 mice treated with either 2 mg of anti- IFN-γ mAb or 2 mg of rat control Ab, at day 3 p.i. with 4,000 sporozoites of *P. yoelii *265BY. C. Parasitaemia of B6 mice treated with 2 mg of anti- IFN-γ mAb at day 4 p.i. with 4,000 sporozoites of *P. yoelii *265BY. Regarding their parasitaemia curves, two groups of mice were identified: "high" and "low" parasitaemia. D. Parasitaemia of B6 and B6.IFN-γ^-/- ^mice following injection of 10^6 ^*P. yoelii *265BY pRBCs. Each graph is representative of two independent experiments with 3 to 9 mice per time point. Results are expressed as mean value ± SD. The statistical differences between groups were determined with the Mann-Whitney test (* = p < 0.05).

Then, the physiological relevance of this early burst of IFN-γ in the control of infection was addressed. B6 and B6.IFN-γ^-/- ^mice were infected with sporozoites and their survival and parasitaemia were monitored. As shown in Figure 5A, B6.IFN-γ^-/- ^mice survived the infection as well as B6 control mice. The parasitaemia of B6.IFN-γ^-/- ^mice was significantly lower than that of B6 mice after day 8 of infection. In addition, the duration of parasitaemia was longer in B6. IFN-γ^-/- ^mice than in control animals, as parasites were cleared from the blood only at day 32 p.i., compared to day 25 in B6 mice (Figure 5A).

To analyse more precisely the consequence of the early peak of IFN-γ on the control of infection, a single injection of anti-IFN-γ mAb was performed in B6 mice, at day 3 or day 4 p.i., and their survival and parasitaemia were monitored. As shown in Figure 5B, B6 mice treated at day 3 p.i. with anti-IFN-γ mAb survived the same as control mice which received irrelevant rat IgG, but their parasitaemia rose significantly slower (since day 6 of infection), and their peak of parasitaemia was significantly lower than that of B6 control mice. However, in contrast to B6.IFN-γ^-/- ^mice, B6 mice treated with anti-IFN-γ mAb at day 3 p.i. eliminated blood stage parasites at the same time as the B6 mice which received irrelevant rat IgG (Figure 5B).

It is also interesting to note that B6 mice, which received anti-IFN-γ mAb on day 4 of infection behaved differently. Indeed, from three independent experiments, 36.8% (7 out of 19) of the mice behave like B6 mice treated at day 3 p.i. ("low parasitaemia" group; Figure 5C), and the other mice displayed parasitaemia similar to that of B6 control mice ("high parasitaemia" group; Figure 5C). Ab treatment at days 5 and 7 p.i. had no effect on the parasitaemia of B6 mice. This shows that the decrease in parasitaemia, observed between day 6 and day 20 p.i., is a consequence of the neutralization of IFN-γ at days 3–4 p.i., and is not due to persistent anti-IFN-γ mAb, which would have neutralized subsequent production of IFN-γ.

Altogether, these results clearly show that, in B6 mice, IFN-γ is not essential to cure of *P. yoelii *primary infection and to eliminate blood-stage parasites, but in its absence, parasitaemia is delayed and lowered. In other models of non-cerebral malaria, such as *P. chabaudi *infection, mice are infected with pRBCs. As the results presented here were unexpected compared to the ones observed in the well-described *P. chabaudi *model [8,9], the hypothesis that the parasite stage used for infection could explain these discrepancies was tested. To clarify this point, B6 and B6.IFN-γ^-/- ^mice were infected with *P. yoelii *pRBCs and their survival and parasitaemia were monitored. As shown in Figure 5D, B6.IFN-γ^-/- ^mice infected with pRBCs behave in the same way as when infected with sporozoites: they survived the infection, their parasitaemia was significantly lower after day 9 of infection, and it lasted longer than that of B6 mice. This shows that the results obtained are not related to the parasite stage used to initiate the infection.

### The early burst of IFN-γ is induced by the pre-erythrocytic stage of *P. yoelii *infection

Finally, in order to determine the parasite developmental stage that induces the early peak of IFN-γ, B6 mice were infected with *P. yoelii*-infected RBCs and the concentration of IFN-γ was determined in their sera. No significant increase of IFN-γ concentrations was observed in the sera of mice at days 3, 4, 5, 6 and 7 post-injection of pRBCs (two independent experiments). Moreover, the level of IFN-γ stayed at the level found in non-infected animals, excepted for two mice out of 36 infected, which showed a small increase of IFN-γ in their serum (one out of five day-3 mice had 4.98 ng/ml of IFN-γ, and one out of nine day 6-mice had 6.96 ng/ml of IFN-γ). These results clearly indicate that the early IFN-γ burst is induced by the pre-erythrocytic stage, and not by the blood-stage.

## Discussion

The data presented here show that the immune response of B6 mice to a *P. yoelii *primary infection initiated with sporozoites is characterized by an early burst of IFN-γ, dependent on the pre-erythrocytic stage.

Somewhat unexpectedly, IFN-γ was not essential to eliminate blood-stage parasites, and moreover, mice controlled their parasitaemia in a more effective manner in the absence of this early burst of IFN-γ. These results are in contradiction with other studies showing that IFN-γ contributes to blood-stage clearance. Indeed, in the well-described *P. chabaudi *model, IFN-γ-deficient mice infected with pRBCs control their parasitaemia less efficiently and die from infection [6,8,9]. This discrepancy cannot be attributed to the parasite stage used to initiate the infection. In addition, it cannot be a consequence of differences in the host genetic background since B6.IFN-γ^-/- ^mice show an increased parasitaemia and exhibit higher mortality following *P. chabaudi *infection [9]. This suggests that the parasite species likely accounts for these differences. In accordance with this latter hypothesis and also with the results presented here, a recent report showed that the control of the primary wave of *P. yoelii *17× parasitaemia is independent of IFN-γ [13].

As the parasitaemia was lower in the absence of IFN-γ, this suggests that IFN-γ would either facilitate *P. yoelii*'s replication, and/or disadvantage its control by the immune system. One possibility could be that IFN-γ favours the parasitaemia through promoting the production of *P. yoelii*'s blood stage target cell, namely the reticulocyte. Further investigations are required to address precisely the mode of action of IFN-γ on the erythrocytic stage in this model.

It is interesting to note that both the constitutive genetic deficiency in IFN-γ and the *in vivo *neutralization with anti-IFN-γ mAb delay the course of parasitaemia and decrease the level of the parasite load. However, only the constitutive deficiency of IFN-γ makes the parasitaemia last longer. This latter observation is reminiscent of the one made in B6 mice infected with *P. chabaudi *pRBCs, showing that IFN-γ favours the appropriate antibody response required to eliminate efficiently the blood-stage parasites after day 20 of infection [24,25]. The results presented here thus argue in favour of two opposite roles played by IFN-γ during *P. yoelii *primary infection in B6 mice: in the first days following sporozoites injection, IFN-γ would be deleterious for the host as it favours the parasitaemia, while, after the first week of infection, it would be beneficial for the host, helping to clear efficiently the blood-stage parasites. In addition, regarding the role of IFN-γ early in infection, only the injection of anti-IFN-γ Abs in B6 mice at day 3 p.i., two days before the cytokine increases in the serum, lowered the parasitaemia in all the treated mice. Injection of the same concentration of antibody performed at days 5 or 7 p.i. had no effect on the parasitaemia, and injection at day 4 p.i. lowered the parasitaemia of approximatively half of the treated mice. These results show that early immune events, occurring in a very narrow window of time are crucial for the control of the subsequent growth of *P. yoelii *in B6 mice. Moreover, these data reflect the importance of the timing and quantity of the release of an inflammatory mediator, i.e. in appropriate amounts at appropriate time during the inflammatory response [26].

Regarding the cell types involved in this early burst of IFN-γ it appears that conventional splenic CD4 T cells are likely to be the main source of this cytokine at day 5 p.i.. These results are coherent with observations made in infected B6.RAG2^-/- ^mice (lacking T and B cells), which showed no early peak of IFN-γ and exhibited a lower parasitaemia than B6 mice following injection of *P. yoelii *sporozoites [11]. Nevertheless, the first cells to produce IFN-γ are innate NK, NKT and γδT cells, which responded simultaneously, suggesting their concomitant activation. Further investigations are required to determine whether this IFN-γ response by innate and CD4 lymphocytes depends on the pre-erythrocytic stage only, or both the pre-erythrocytic and the erythrocytic stages.

It is also interesting to note that CD1d-restricted NKT cells are not necessary for the initiation of this early production of IFN-γ. Together with previously published results showing that CD1d-independent NKT cells are induced in the spleen of *P. yoelii*-infected B6 mice and are biased towards the production of Th1-type cytokines [12], these data strengthen the idea that CD1d-independent NKT cells are involved in *P. yoelii*-induced early Th1 immune response.

The concentration of IFN-γ in the blood rapidly decreased. This could be due to its capture by target receptors and/or to a quick shut down of its production by regulatory mechanisms, such as TGF-β and/or IL-10 production [13,27].

Finally, this early burst of IFN-γ was induced by the pre-erythrocytic stage, showing the impact of this latter one on the immune response taking place during the subsequent blood stage.

## Conclusion

Overall, the results presented here argue in favour of two opposite roles for IFN-γ during *P. yoelii *primary infection in B6 mice and show that an early IFN-γ response can be deleterious for the host regarding the control of parasite growth. Taken together with the recent report published by Couper *et al *[13], these data challenge the prevailing idea that IFN-γ is an essential mediator of protection in malaria. These results also support the idea that the commitment towards protection or pathology takes place very early after infection since we show that precocious immune events impact on the control of the parasite's growth much later in infection.

Finally, these data highlight the complex regulation of the primary immune response to *P. yoelii *sporozoite-induced malaria, since it is shown that the pre-erythrocytic stage induces an early burst of IFN-γ that, directly or indirectly, favours parasitaemia. These results also strenghten the need for studying the immune response to *Plasmodium *in models closest as possible to the physiology of the natural infection.

## Competing interests

The authors declare that they have no competing interests.

## Authors' contributions

VS, JR and SP designed the study. VS and JR performed the experiments and wrote the manuscript. OG performed the statistical analyses. EB performed some of the ELISA experiments. PAC and SP helped to write the manuscript. All authors read and approved the final manuscript.
